# mNP hyperthermia and hypofractionated radiation activate similar immunogenetic and cytotoxic pathways

**DOI:** 10.1080/02656736.2020.1802070

**Published:** 2020

**Authors:** Kayla E. A. Duval, James D. Petryk, Margaret A. Crary-Burney, Eugene Demidenko, Robert J. Wagner, P. Jack Hoopes

**Affiliations:** a Thayer School of Engineering, Dartmouth College, Hanover, NH, USA; b Geisel School of Medicine, Dartmouth College, Hanover, NH, USA

**Keywords:** Magnetic nanoparticle hyperthermia, immunology, radiation, apoptosis

## Abstract

**Objective:**

The goal of this study is to better understand the immunogenetic expression and related cytotoxic responses of moderate but clinically relevant doses of hypofractionated radiation (1×15 Gy and 3×8 Gy) and magnetic nanoparticle hyperthermia (mNPH, CEM43 30)

**Methods:**

Genetic, protein, immunopathology and tumor growth delay assessments were used to determine the immune and cytotoxic responses following radiation and mNPH alone and in combination. Although the thermal dose used, 43 C°/30 min (CEM43 30), typically results in modest independent cytotoxicity, it has shown the ability to stimulate an immune response and enhance other cancer treatments. The radiation doses studied (15 Gy and 3×8 Gy) are commonly used in preclinical research and are effective in selected stereotactic and palliative treatment settings, however they are not commonly used as first-line primary tumor treatment regimens.

**Results:**

Our RNA-based genetic results suggest that while many of the cytotoxic and immune gene and protein pathways for radiation and hyperthermia are similar, radiation, at the doses used, results in a more consistent and expansive anti-cancer immune/cytotoxic expression profile. These results were supported by immunohistochemistry based cytotoxic T-cell tumor infiltration and tumor growth delay studies. When used together radiation and hyperthermia led to greater immune and cytotoxic activity than either modality alone.

**Conclusion:**

This study clearly shows that modest, but commonly used hypofractionated radiation and hyperthermia doses share many important immune and cytotoxic pathways and that combining the treatments, as compared to either treatment alone, results in genetic and biological anti-cancer benefits.

## Introduction

Cancer patients are living longer, however cancer incidence and related deaths continue to rise [1]. As cancer clinicians and researchers learn more about cancer treatment biology and efficacy, it is clear that multimodality therapy remains necessary and that stimulating an anti-cancer immune response has life-saving potential in some situations. Some longstanding cancer treatments, such as hyperthermia, that were originally believed to be effective only as independent tumor ablative therapies or as adjuncts to radiation therapy and/or chemotherapy, are now showing the ability to be potent cancer immune stimulators at doses considered safe in most settings. Although hyperthermia has been studied as a cancer treatment for centuries, the most useful hyperthermia doses and delivery techniques, including microwave, radiofrequency, focused ultrasound, laser and heatable nanoparticles, are still being debated for individual disease and disease sites [2–8]. The hyperthermia delivery technique studied here, magnetic nanoparticle hyperthermia (mNPH), works by delivering magnetic nanoparticles to a tumor site then exposing the tumor region to an alternating magnetic field (AMF) for a specific length of time. This combination results in intracellular and/or extracellular heating of the targeted tissue. As with other hyperthermia techniques, the level of thermal dose dictates the biological effect [9]. Typically, the higher and longer the temperature rise the greater the cytotoxicity, however there may be unexplained benefits of intracellular mNP hyperthermia. Like other cancer therapeutics, the tumor-normal tissue therapeutic ratio for hyperthermia must always be considered. In this study we delivered a constant thermal dose of 30 CEM 43 °C to the tumors of all subjects. CEM 43 is defined as cumulative equivalent minutes at 43 °C and has been used extensively to describe treatments that contain complex thermal histories [10]. The dose we used in this study has been shown to produce significant immune responses in previous studies [11].

The immune potential of radiation and hyperthermia is the topic of many current studies. There is, however, significant controversy regarding the effect of total dose and fractionation, for both modalities [12,13]. While high doses, of either modality, resulting in acute cell death, initiate a robust general inflammatory response, lower doses may, according to many investigations, initiate a more subtle pathological response (monocyte/macrophage based response). This response may have an even greater immune potential than the larger ablative/necrotic doses [14].

Radiation therapy has conventionally consisted of many small doses over a one month period (2 Gy × 30 treatments). Pathological examination of the tumor and irradiated normal tissues typically showed a modest inflammatory or immune response, unless a significantly toxic (necrotic) dose was delivered. In recent years, the delivery of larger fewer radiation doses (hypofractionated radiation) has begun to be used in some tumor sites/locations [15–18]. This treatment scheme may lose some of the inherent therapeutic ratio advantages (tumor vs normal tissue) of small doses per fraction but affords others such as targeting, dosimetry, treatment and potentially immune activation [13,19–21]. In addition to the implementation of fewer, larger doses (hypofractionated radiation), there is also a significant increase in the use of a single large dose treatment schemes (stereotactic radiation) [17,22]. In this study, we chose to compare a 3 × 8 Gy treatment regimen and a single 15 Gy regimen. These doses have similar BED/EQD2 values.

Evidence of immune effects, following radiation and magnetic nanoparticle hyperthermia, in our own laboratory and from other investigators, led us to hypothesize that combining radiation with mNPH will enhance the tumor immune response and lead to improved tumor growth delay. We used genomic and cell infiltration responses to better understand the immune effect of hyperthermia alone and in combination with hypofractionated radiation. The overall hypothesis and aim of this study was that analysis of post treatment genetic effects, immune cell infiltration of the tumor, and tumor growth delay would elucidate the anticancer immune response of CEM43 30 hyperthermia, and the further enhanced effect of this dose of hyperthermia with hypofractionated radiation. Our survey of hundreds of genes allows us to provide robust expression data that can be used to inform future studies with potential additional biological targets.

## Methods

### Mouse tumor model

In this study we have exclusively used B16F10 murine melanoma cells (American Type Culture Collection (ATCC), Manassas, VA). The B16F10 cells were cultured in RPMI media with 10% fetal bovine serum, and 1% antibiotics until inoculation. Tumors were initiated in 6-week-old C57BL6 female mice (Charles River) by injecting 2 × 10^6^ B16F10 cells intradermally in the right flank. Following implant, tumors were measured daily and placed on study when they reached 110 mm^3^ (±20 mm^3^). Based on information from our own previous studies and the literature we determined that the five following groups, control, 3×8Gy, 15 Gy, mNPH, mNPH + 3 × 8 Gy, and mNPH + 15 Gy, created a translationally effective evaluation and comparison.

### Magnetic nanoparticle hyperthermia

Magnetic nanoparticle hyperthermia has been commonly used over the past 20 years in cancer treatment [23–25]. All mNPH experiments used 100 nm ± 30 nm (hydrodynamic diameter) dextran coated matrix iron oxide nanoparticles (Bionized NanoFerrite/BNF micromod Partikeltechnologie GmbH, Rostock, Germany) suspended in H_2_O, 75–80% (w/w). The stock nanoparticle concentration in solution was 45 mg/ml (24.5 mg/ml iron). Nanoparticles were injected intratumorally at a dose of 7.5 mg of iron per gram (cm^3^) of tumor (30–40 μl for the average 110 (±20) mm^3^ tumor). Three hours post injection, tumors underwent alternating magnetic field (AMF) exposure. The AMF was generated by a water cooled, whole body circular coil (Fluxtrol Inc., Auburn Hills, MI) powered by a Huttinger TIG 10/300 generator (Freiburg, Germany) tuned to operate at 165 kHz. Mouse rectal temperatures were monitored and recorded throughout the hyperthermia treatment using 0.3 mm diameter FISO fiber optic probes (FISO Inc., Quebec, Canada) at a frequency of 1 Hz. The mouse rectal temperature was maintained at 37 °*C* ± 1 °C by a modulating a thermal air jacket which surrounded the mouse containment and gas anesthesia vessel. Tumor temperatures were monitored using a triple sensor FISO probe (3 mm sensor separation) implanted entirely within the tumor parenchyma, allowing for monitoring of 3 equally spaced locations along the center axis of the tumor. The assessment of multiple simultaneous temperatures is important to diminish the effect of heterogenous tumor heating. The magnetic field was turned off once a CEM43 30 (43 °C/30 min) was reached. Field strength was continuously controlled to ensure the tumor temperature was maintained between 43–44 °C.

### Radiation therapy

A Varian linear accelerator was used to deliver 6 MeV electron doses of 8 Gy or 15 Gy at a dose rate of 8 Gy/minute. A precise custom designed collimator allowed for a uniform dose delivery to the entire tumor volume and a 2 mm peritumor region. The SSD (source to surface distance) was 100 cm. Radiation was given three times on consecutive days for the 8 Gy/fraction treatment. When mNPH was employed, radiation was started 4 h after magnetic nanoparticle injection, and immediately followed by a single hyperthermia treatment. Unpublished data from our lab indicates optimal mNP uptake between 3–4 h.

### Post-treatment endpoints

Two treatment endpoints were utilized. The first time endpoint cohort, 110 h (± 6 h) post treatment, provided RNA for genetic analysis (NanoString) and tissue for immunohistochemical sections that allowed for quantification of selected T cell populations, i.e., cytotoxic CD8+ cells. The second cohort was entered into a 3-fold (3x) tumor growth delay assay for determination of treatment efficacy, where the endpoint was time, in days, to reach 3x treatment volume.

### Immunohistochemistry

Following sacrifice and tumor removal, the tumors were bisected. One half was processed for RNA and protein analysis, the other half for H&E and immunohistochemical microscopy slide staining. The histology sections allow for segmental but reasonable representation of the mid and lateral portions of the tumor. Although not a comprehensive global assessment of immune cell infiltration, historical experience suggests this level of spatial assessment provides a meaningful assessment of overall immune cell infiltration into the tumor. Two sets of slides were prepared: conventional H&E for morphological assessment and CD8+ antibody (Cell Signaling) staining assessment of cytotoxic T cell (CD8+) infiltration. The different staining parameters (separated spatially by 4–8 micron) allowed for effective co-registration of morphological change and CD8+ infiltration and immune initiated tumor cell death (ICD). Quantification of CD8+ infiltration was accomplished using 40x magnification, and a 100 crosshair grid was placed on each field of view to aid in quantification of the relative number of CD8+ cell per high power field. Twenty randomly determined fields per tumor were assessed. The assessment of CD8+ cell infiltration was presented in the context of the percentage of the CD8+ cells with respect to the total number of cells in each field of view.

### RNA collection and analysis

RNA was isolated from tumors using the Qiagen RNeasy Mini Kit, and quantified and normalized across samples. Samples were prepared and mRNA expression quantified using the NanoString PanCancer Murine Immune Profiling Panel, with an additional custom panel of cell cycle, apoptosis, and DNA damage repair genes, and the NanoString nCounter Analysis System. Results were analyzed with nSolver Analysis (v. 4.0) and Advanced Analysis Software (v. 2.0). *N* = 4–5 tumors per treatment.

### Tumor growth/control

To assess hyperthermia and/or radiation treatment effect in the tumor, mice were placed on study when tumors reached 110 mm^3^ (±20mm^3^). This volume was typically achieved 8–12 days post inoculation. Tumors were measured daily/5 times per week, in 3 dimensions using a length−*width*depth*pi/6 formula to calculate tumor volume. Treatments were randomly assigned, and measurements were completed in a blinded fashion, without knowing the treatment. Mice were removed from the study when their tumors reached 3x treatment volume. Kaplan-Meier and tumor growth curves were used to assess this result. Rate of tumor growth for each treatment was determined by taking the average of each animals tumor growth rate using an exponential model of tumor growth, using the first and last tumor measurements recorded.

### Statistical analysis

Genetic/RNA (nanoString) analysis utilized XQuartz and R statistical software *via* an nSolver Advanced Analysis software package to carry out these statistical tests in the differential expression module. For the tumor growth delay study, MATLAB was used to carry out ANOVA tests for comparison of all treatment groups in terms of survival days and rate of tumor growth. An unbalanced two-way ANOVA was used with hyperthermia (two levels) and radiation (three levels) as the factors. This allows for determining if there was significant difference between radiation groups, between hyperthermia or no hyperthermia, and if these two factors (hyperthermia and radiation) have an interaction effect. The multcompare function in MATLAB was then used with the ANOVA stats output to determine which treatments were statistically different from each other. Statistical significance is defined as *p*-value < .05.

## Results

The hypothesis underlying this study was that a commonly used adjuvant hyperthermia dose (CEM43 30) would stimulate an antitumor immune and/or cytotoxic response that utilized different immune and cytotoxic genetic pathways than a commonly used preclinical and clinical hypofractionated radiation dose (15 Gy or 3 × 8 Gy). Although not a primary goal we also studied the possibility that a combination of hyperthermia and radiation would stimulate a greater response than either modality alone and that an efficacy study would confirm the genetic responses. Additionally, the combinatorial effect would vary with a hypofractionated versus large single dose treatment. These effects were investigated through quantitative RNA expression, immunohistochemistry of cytotoxic cells, and tumor growth delay.

### NanoString mRNA expression results

**Figure 1.**
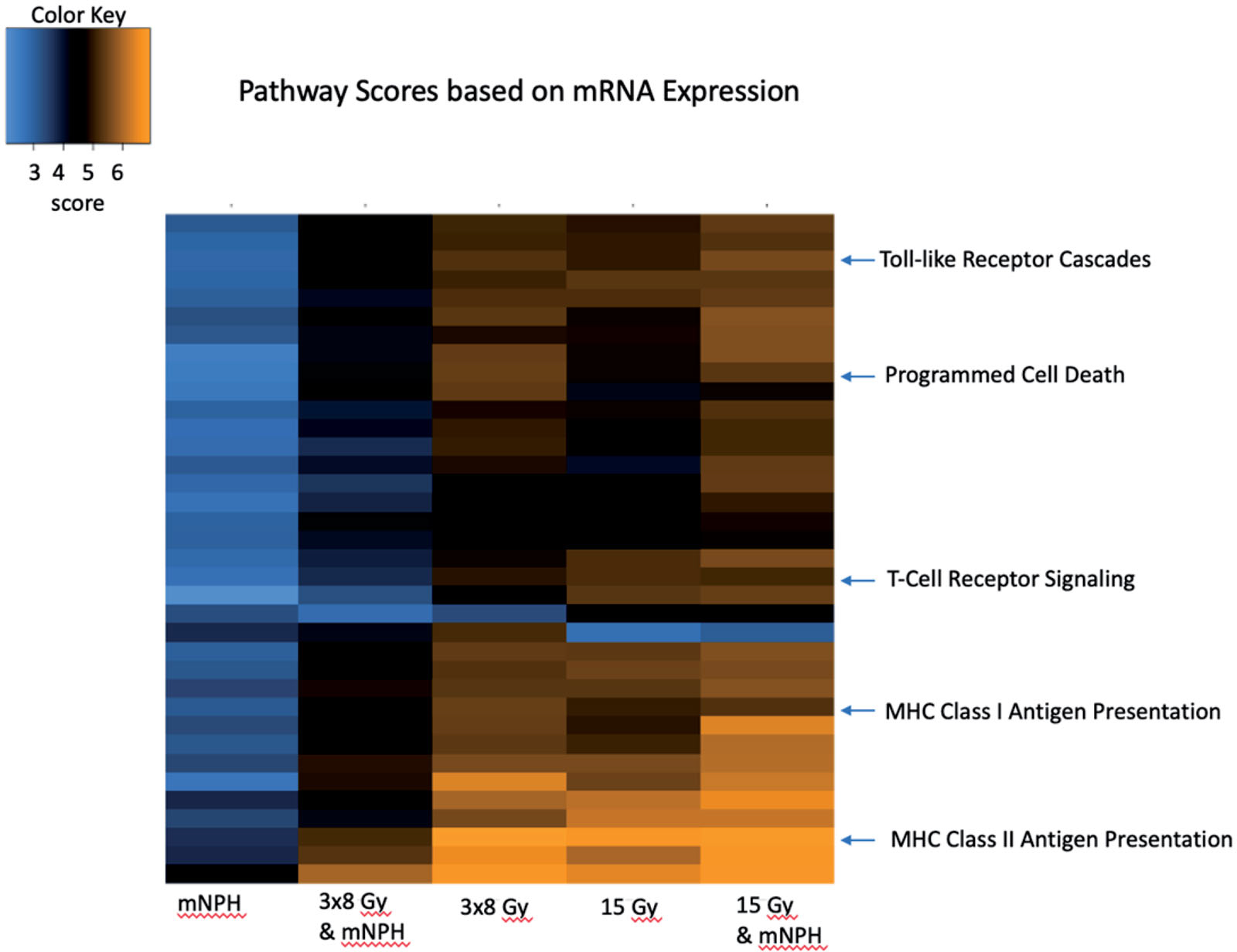
This heatmap demonstrates genetic pathway scores for the various treatment groups. This graph depicts the information for many pathways, but highlighted here are important immune and cytotoxic signaling pathways. There are clear differences in pathway scores (determined by cumulative expression for all genes in the pathway) in treatments involving radiation, with 15 Gy mNPH having more changes as compared to 3×8 Gy mNPH. The heatmap colors represent different pathway scores ranging from lower (blue) to higher (yellow).

Compared to control, a number of important immune and apoptotic genes and pathways were upregulated across all treatment groups. The most notable genes appear in Table 1 for clearer representation of expression changes across treatment cohorts. The common and important pathways discussed, and their expression levels for each treatment, are depicted in the heatmap in Figure 1 using pathway scores. NanoString pathway scores are derived from the expression levels of all the genes in a pathway, using the first principal component; if most genes have increased expression the pathway score will depict that. The pathways of note are indicated, with higher positive expression levels demonstrated in yellow, and lower expression levels in blue.

### mNPH versus control

mNPH altered the expression of a number of important immune and apoptotic genes and pathways. Notable were: natural killer cell mediated cytotoxicity (receptors ITGAL, ITGB2, and NKG2D) [26]; antigen processing/presentation (various MHCI and MHCII genes leading to CD8 on T cells [27], and KIR on NK cells [26]); and T cell receptor signaling pathways (ICOS) [28]. Additionally, pro-apoptosis genes, in the intrinsic and extrinsic pathways, as well as the T cell mediated apoptotic pathways were upregulated (e.g., Fas, Bim, NOXA, GZMB, and Perforin [29]). However, several anti-apoptosis/pro-survival genes were also upregulated (such as Gadd45 [29]), as well as NK cell inhibitory receptors (CD94, and NKG2A/B [30]), and T-cell inhibitory receptors (PD-1, and CD45 [31]). In summary, the data demonstrates that adjuvant level mNPH engages many important cytotoxicity and apoptosis genetic pathways. While the majority of these genes and pathways are pro-immune and pro-apoptotic it is noted that several, such as MCL1, Bcl6, IL-7R, and SOCS are believed, in some situations to be immune regulatory and/or pro-tumor survival [32].

### Radiation versus control

Like mNPH, 15 Gy and 3 × 8 Gy resulted in numerous gene expression changes in the immune and cytotoxic pathways. Notable, were increases in natural killer cell mediated cytotoxicity pathway (including activating receptors ITGAL, ITGAB2, CD94, NKG2D); the immune cell mediated apoptotic pathway (including perforin, granzymes, FAS, TRAIL, Caspase 3 [33]); the toll-like receptor pathway (including T cell stimulation factors CD40, CD80, CD84) and the chemotactic effectors MIG, RANTES, and TLR1,2,6,7,8 and 9 [34,35]. These genes are all known to stimulate an antitumor cytotoxic effect through immune mediated and/or apoptotic cascades. Consistent with mNPH, 15 Gy and 3 × 8 Gy also enhanced expression of important pro-survival genes (Gadd45, Bcl-XL, and Bcl-2).

### mNPH ± radiation versus control

In general the combination of mNPH and radiation (15 Gy or 3 × 8 Gy) led to higher expression of most important immune and cytotoxic genes as compared to either modality alone. Natural killer cell mediated cytotoxicity, antigen processing/presentation, and T cell receptor were the most prominently expressed pathways. The inhibitory receptors seen with mNPH such as PD-1, CD45, CD94, NKG2A/B were also upregulated with the combination treatment. In addition, the combination increased the expression of other inhibitory receptors such as CTLA4 and Ly49G2. Similar to mNPH and radiation alone, combined therapy also activated apoptosis pathways and several pro-survival genes, such as Gadd45 and Bcl-XL. Gene expression levels are demonstrated in Table 1 for all treatment cohorts.

### mNPH±radiation versus mNPH

The combined mNPH + radiation treatments resulted in the increased expression of a majority of notable immune and cytotoxic genes compared to adjuvant hyperthermia alone. However, there were differences depending on the radiation dose. When 3 × 8 Gy was combined with mNPH, only one gene (Ncr1) was differentially expressed with greater than 2-fold change when compared to mNPH alone. Compared to mNPH alone, 15 Gy + mNPH differentially expressed Ncr1 as well as additional genes in the natural killer cell mediated cytotoxicity pathway. More than 80 genes were differentially expressed following 15 Gy + mNPH as compared to mNPH alone. Many of these genes/pathways were associated with immune stimulation and infiltration, including natural killer cell mediated cytotoxicity, TLR based chemotactic effects, T cell stimulation, and leukocyte transendothelial migration.

### mNPH ± radiation versus radiation alone

The combination of mNPH + radiation treatments resulted in minimal expression differences for most pathways compared to radiation alone. Pathways with minimal differential expression included antigen presentation, immune cell cytotoxicity, and apoptosis. However, when comparing to 15 Gy to 15 Gy + mNPH, the combined therapy demonstrated increased expression in the cell adhesion molecules pathway, including CD31, CD34, Cdh5, Tie1, and Il1b [36]. These differences, except for Il1b, were only seen with the combined mNPH +15 Gy dose. Interestingly l1b was increased with 3 × 8 Gy + mNPH to 3 × 8 Gy alone. Finally, there were modest increases following 3 × 8 Gy + mNPH vs 3 × 8 Gy in the T cell receptor signaling pathway, and cytokine-cytokine receptor pathways.

### Immunohistochemistry assessment of CD81 infiltration of tumors

**Figure 2.**
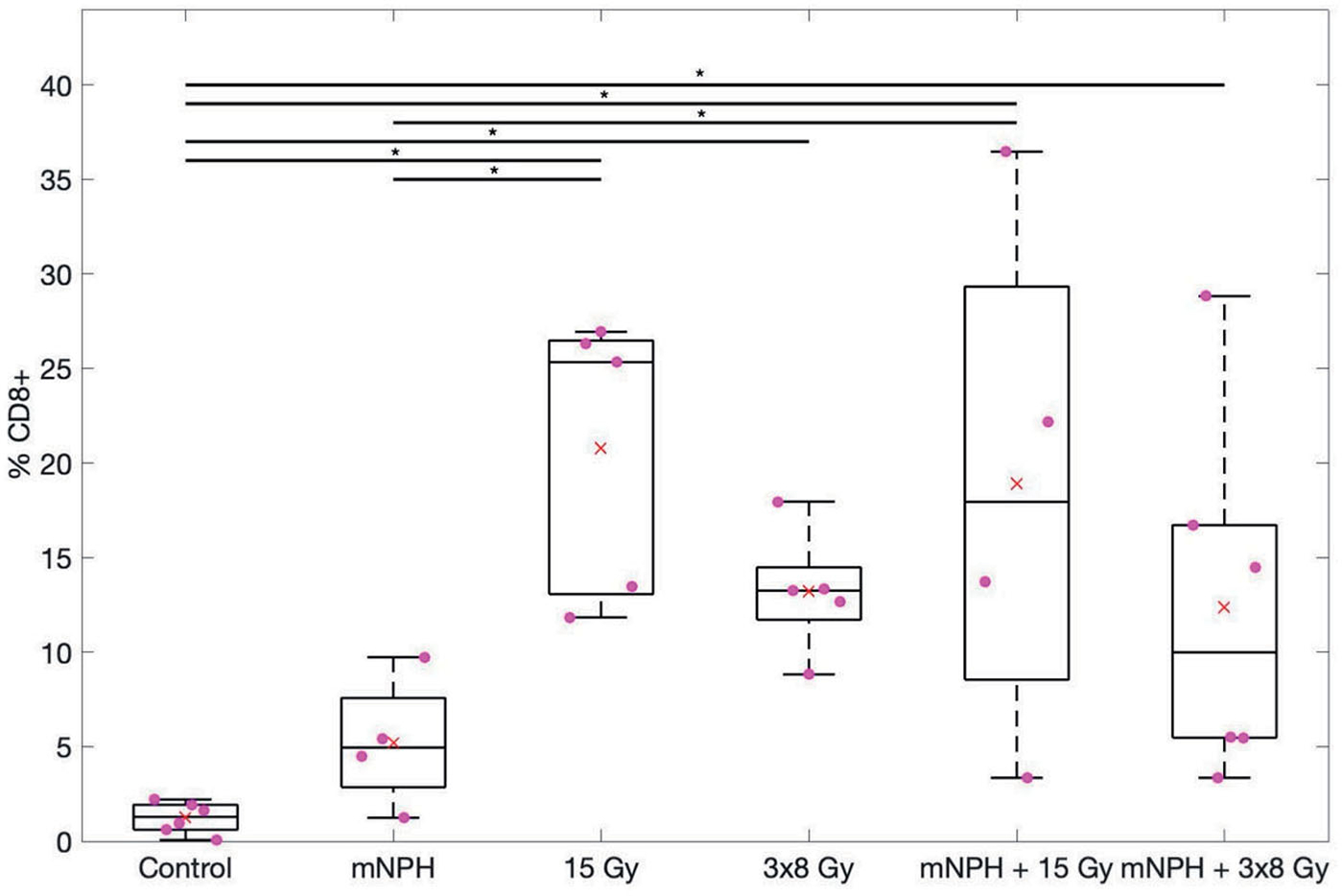
This box and whisker graph illustrates the CD8 population percentages for each treatment. The combinatorial treatments have a greater spread than the radiation alone groups, and 15 Gy alone and in combination leads to greater CD8 populations within the tumor. The red × demonstrates the mean, and the * means *p<.05.

CD8+ cells are most often cytotoxic T cells, or a small subset of natural killer cells, indicative of an antitumor cytotoxic immune response. Using CD8+ immunohistochemistry and a morphometric point count technique [37] we determined the relative level of CD8+ infiltration into control or treated tumors (Figure 2). Control tumors had a CD8+ population of 1.23% (SEM = 0.3). Hyperthermia alone resulted in a CD8+ population of 5.22% (SEM = 1.8). Radiation treatment of 3 × 8 Gy alone led to a CD8+ population of 13.22% (SEM = 1.5), while 15 Gy alone led to a population of 20.78% (SEM = 3.3). 3 × 8 Gy + mNPH led to a wide range of CD8+ infiltration depending on the tumor, ranging from 3% up to 29%, with an average of 12.4% (SEM = 4.0). 15 Gy + mNPH also led to a wide range of CD8+ infiltration (3.4% to 36%), with an average of 18.94% (SEM= 7.0). Statistical analysis revealed only the 15 Gy radiation cohorts were significantly different than control, with no other groups having significant differences.

### Tumor growth delay

**Figure 3.**
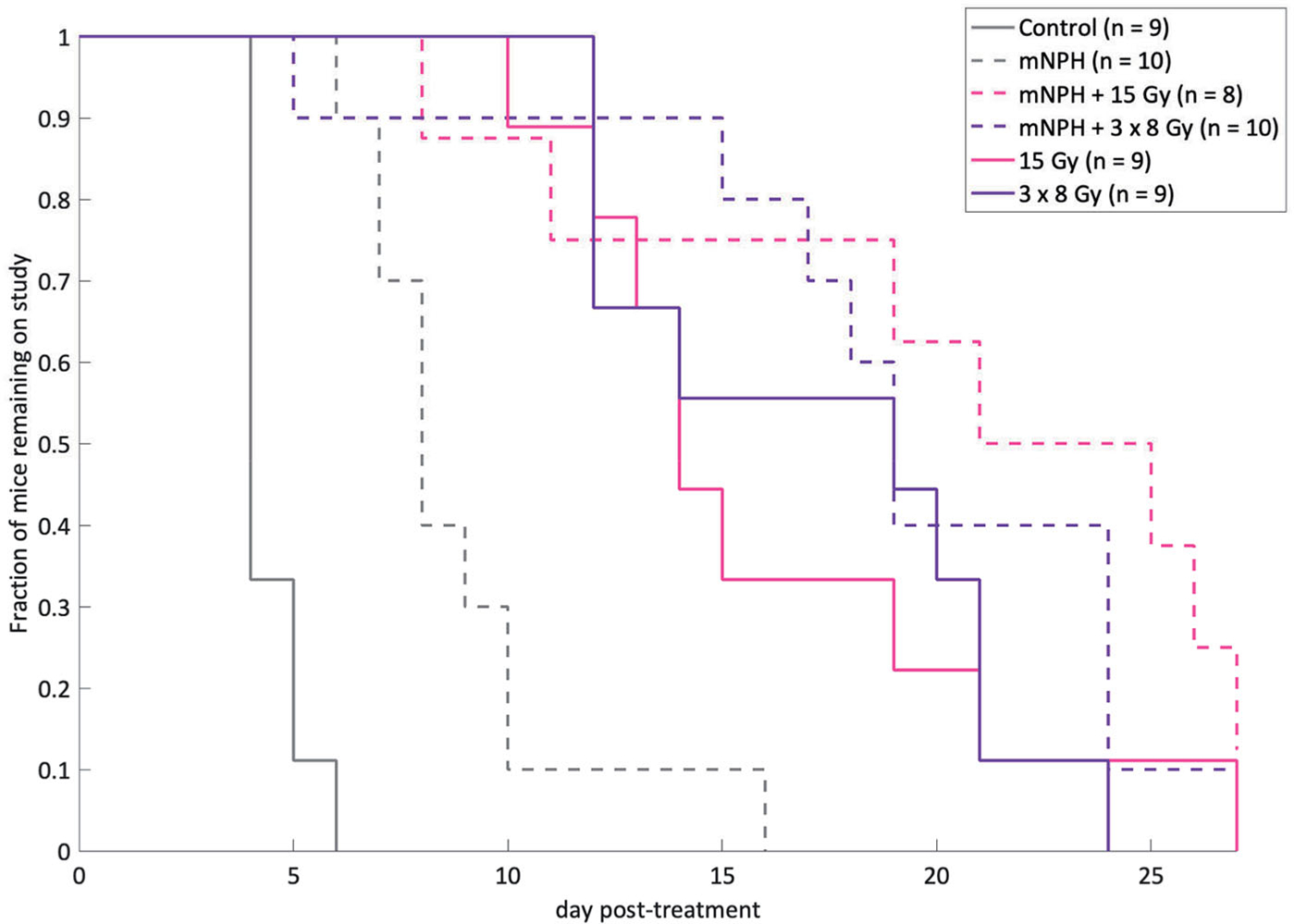
This graph demonstrates post-treatment tumor response, for the following groups: control, mNPH, 15 Gy, 3×8Gy, 15 Gy + mNPH, and 3×8 Gy + mNPH. All treatments resulted in statistically significant improvements as compared to control. The mNPH treatment, which is generally considered minimally cytotoxic was expectedly less effective than either radiation dose or the combination.

Although we do not consider tumor control to be a major endpoint of this study, we performed a tumor growth delay study, to if the gene expression changes and CD8+ counts are associated with differences in tumor growth delay. Tumors were measured using calipers at least five times a week, with volumes calculated using the previously described technique. Each study arm contained *n* = 8–10 animals. Animals were removed from the study when tumors reached 3x the starting volume. Untreated control mice remained on study an average of 4.4 days. Mice treated with mNPH, 15 Gy, 3 × 8 Gy, or a combination (15 Gy or 3 × 8 Gy) remained an average of 8.9, 16.1, 17.2, 21.5, and 20 days post-treatment respectively (Figure 3). There was a statistical difference (*p* <.05) between control and all radiation groups, and between mNPH and all radiation groups except 15 Gy alone (*p*=.08 for mNPH versus 15 Gy). The ANOVA assessment showed the tumor growth delay significance of hyperthermia and radiation alone and in combination vs control and for radiation and the combination vs hyperthermia but not for the combined treatment vs radiation.

**Figure 4.**
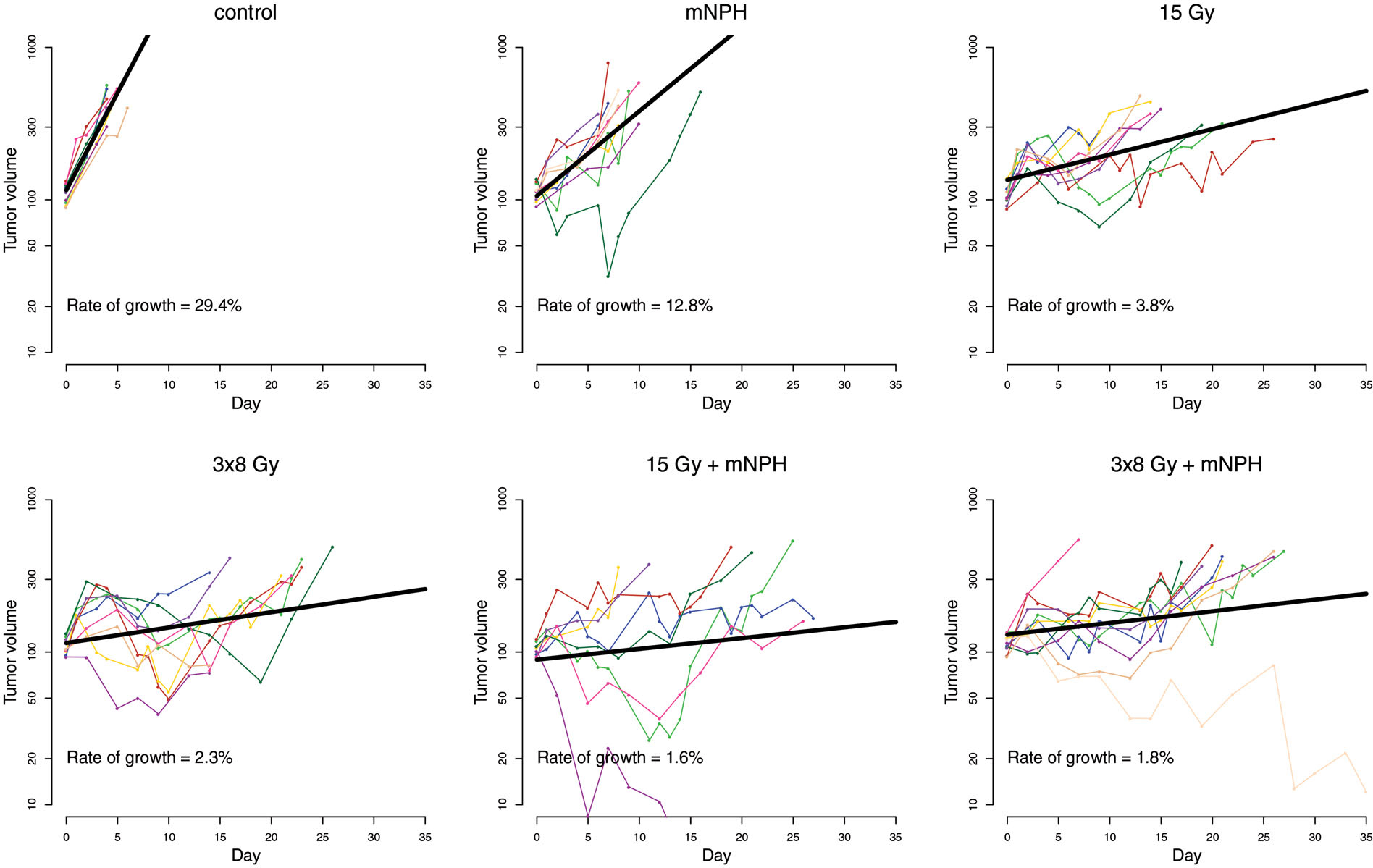
This graph demonstrates the tumor growth curves for all animals in each treatment cohort, with tumor volume on a log scale. The average growth rate is reported for each treatment. Each treatment led to a decreased rate of growth as compared to control.

In addition to time on study, tumor growth was looked at as shown in Figure 4. The growth rate for each animal was calculated using a mixed effect model [38,39]. The average rates of growth (% volume change per day) for the cohorts were: control = 29.4%, mNPH = 12.8%, 15 Gy= 3.8%, 3 × 8 Gy = 2.3%, 15 Gy + mNPH = 1.6%, and 3 × 8 Gy mNPH = 1.8%. The rate of growths for all treatment cohorts were statistically significantly lower than control, with the combination of radiation and hyperthermia leading to the lowest rates of growth. All of the radiation cohort’s growth rates were significantly different than mNPH, and 3 × 8 Gy was significantly lower than 15 Gy. The rate of growth in tumors treated with the combination treatment with 15 Gy was significantly lower than 15 Gy alone, however the same was not true for 3 × 8 Gy + mNPH compared to 3 × 8 Gy. Finally, there was not a significant difference bet ween 15 Gy + mNPH and 3 × 8Gy + mNPH.

## Discussion

Magnetic nanoparticle hyperthermia (CEM43 30) and hypofractionated radiation (15 Gy or 3 × 8 Gy) have been shown to be immunogenic and when appropriately combined the two treatments can enhance tumor control and the tumornormal tissue therapeutic ratio [11,12]. It is the goal of this study to test the hypothesis that immune activation is partially responsible for the improved tumor growth delay created by combining hypofractionated radiation and modest tumor heating. Although there are many genetic, cell, and growth response assessments of heated and/or irradiated tumors, very few studies have focused on the immune and cytotoxic genetic pathways. Because this information is sparse in the literature, we had limited data on which to build or compare.

**Figure 5.**
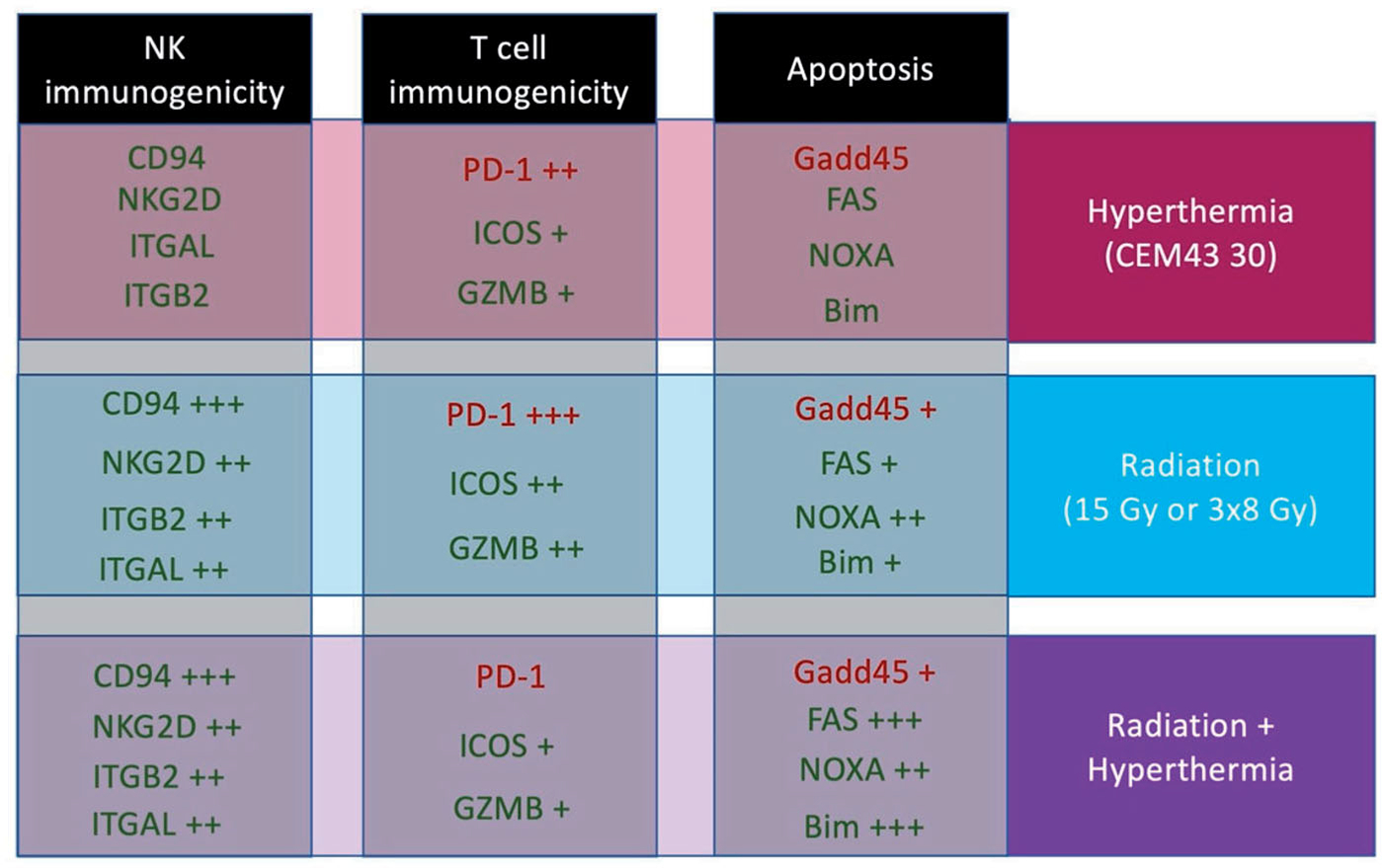
This diagram demonstrates the specific gene expression changes identified in this study. The genes are separated by effect/pathway and treatment type, with green indicating pro-immunogenic or pro-apoptotic genes, and red indicating genes that are anti-immunogenic or anti-apoptotic. The plus signs (+) corresponds to the level of increased gene expression, as compared to control. It is important to note that the combination of hypofractionated radiation therapy with magnetic nanoparticle hyperthermia led to greater changes in both Natural Killer cell and T cell activation/cytotoxicity, as well as apoptosis.

We demonstrated that mNPH induces meaningful immune stimulatory signals, most significantly in the natural killer cell [40], T-cell activation [26–28] and apoptotic pathways [29,33]. Similarly, hypofractionated radiation and the combination with hyperthermia demonstrated increased immune signaling largely in the same pathways, as summarized in Figure 5. It should be noted, that although the tumor growth curves for radiation alone and the combination of radiation and hyperthermia had similar patterns, there were several animals in the combination therapy groups with a greatly enhanced tumor growth delay. Interestingly, our results demonstrate that 15 Gy appears superior to 3 × 8 Gy in combination with hyperthermia, at all levels of assessment, from molecular, through pre-clinical tumor growth delay, regarding the overall immune mediated anti-tumor response. These results lay the groundwork for further mechanistic studies and models to build upon.

As mentioned, our results show that the tested hypofractionated radiation and hyperthermia doses stimulate many of the same important immune and cytotoxic pathways. However, the level of gene enhancement and tumor response for the combination therapy, while increased over either therapy alone, was not as great as anticipated. This finding raises a critical question regarding the basic ability of heat and radiation to create an enhanced anti-cancer effect. If the answer is, based on huge clinical data base, that heat and radiation can work effectively together why is the genetic evidence not clearer in this study. We believe there are two plausible explanations: (1) mNPH and radiation, to great extent, stimulate similar immunogenetic pathways, and this overlapping stimulation obscures any additive or synergistic enhancement we would expect to see. (2) We have studied specific radiation and hyperthermia doses, timing, delivery, and combination parameters. Even slight alterations in these parameters could make significant differences in the genetic and biological effects determined.

From a clinical translation standpoint, the most important finding of this study is that the combination therapy was most effective at stimulating immunogenetic cytotoxic and tumor growth delay effects. Importantly, each aspect we analyzed, from mRNA through tumor growth delay, indicated that 15 Gy rather than 3 × 8 Gy is slightly more effective in combination with mNPH. This report demonstrates the importance of understanding the genetic aspects of immune and cytotoxic anti-cancer treatment for optimization of radiation and hyperthermia combinatorial therapies. Additionally, we feel foundational data, such as that presented here, is necessary to inform future hyperthermia and radiation studies that address a systemic immune response.

## Figures and Tables

**Table 1.** This table demonstrates the relative change (linear fold change in differential expression) for all treatment cohorts as compared to control for genes that are significantly affected by mNPH and/or radiation and discussed in the text.

Gene	mNPH	15 Gy	3 × 8 Gy	15 Gy mNPH	3 × 8 Gy mNPH	Protein & pathway/function

ITGAL	4.03* (1.4)	9.08* (1.4)	7.8* (1.4)	8.98* (1.4)	5.62* (1.4)	Integrin alpha L (CD11a) – natural killer cell mediated cytotoxicity, activating receptor
ITGB2	3.18* (1.3)	6.38* (1.3)	6.03* (1.2)	7.02* (1.3)	5.22* (1.3)	Integrin beta chain-2 (CD18) – natural killer cell mediated cytotoxicity, activating receptor
CD94/(klrd1)	6.03* (1.5)	24.9* (1.4)	14.9* (1.4)	21.9* (1.4)	7.89* (1.5)	Killer cell lectin-like receptor D1(CD94) – natural killer cell mediated cytotoxicity, inhibitory receptor
NKG2D/(klrk1)	10* (1.5)	27.5* (1.5)	26.6* (1.4)	22* (1.5)	14.7* (1.5)	Killer cell lectin-like receptor k1 (NKG2D) – natural killer cell mediated cytotoxicity, activating receptor
ICOS	9.72* (1.7)	30.5* (1.6)	22.2* (1.6)	21.8* (1.7)	16.2* (1.7)	Inducible T-cell costimulator (CD278) – T cell receptor signaling pathway, activating receptor
FAS	3.05* (1.3)	3.95* (1.3)	6.43* (1.3)	4.17* (1.3)	4.48* (1.3)	FAS – apoptosis pathway, death receptor (activating)
Bim	2.31* (1.2)	3.68* (1.2)	2.96* (1.2)	4.2* (1.2)	2.71* (1.2)	Bim (Bcl-2-like protein 11) – apoptosis pathway, apoptosis activator
NOXA	4.14* (1.5)	8.94* (1.5)	9.45* (1.4)	9* (1.5)	9.68* (1.5)	Noxa – apoptosis pathway (Bcl2 family), pro-apoptosis
GZMB	9.87* (1.5)	29.4* (1.5)	31.5* (1.5)	24.7* (1.5)	18.6* (1.6)	Granzyme B – T-cell mediated apoptosis (and some Natural killer cell mediated), apoptosis inducer
Gadd45	1.58* (1.2)	2.38* (1.2)	2.6* (1.2)	2.05* (1.2)	2.79* (1.2)	Gadd45 – DNA Damage pathway, pro-survival/anti-apoptosis
PD-1	16.5* (1.8)	34* (1.8)	26.5* (1.7)	25.2* (1.8)	8.64* (1.8)	Programmed Cell Death 1 – T cell receptor signaling, inhibitory/immune regulatory
CTLA4	1.76 (1.3)	5.16* (1.3)	4.51* (1.3)	3.92* (1.3)	1.96* (1.4)	Cytotoxic T-lymphocyte-associated protein 4 – T cell receptor signaling, inhibitory/immune regulatory
NCR1	1.6 (1.8)	29.2* (1.6)	36.5* (1.6)	53.6* (1.6)	22.5* (1.7)	Natural cytotoxicity triggering receptor 1 (CD335) – natural killer cell mediated cytotoxicity, activating receptor

Average fold change with standard error are reported.

* *p* < .05.

## References

[1] World Health Organization. WHO | Cancer Factsheet. WHO 2018. doi:/entity/mediacentre/factsheets/fs297/en/index.html

[2] Kumar CSSR, Mohammad F. Magnetic nanomaterials for hyperthermia-based therapy and controlled drug delivery. Adv Drug Deliv Rev. 2011;63(9):789–808. 21447363 10.1016/j.addr.2011.03.008 PMC3138885

[3] Grüll H, Langereis S. Hyperthermia-triggered drug delivery from€ temperature-sensitive liposomes using MRI-guided high intensity focused ultrasound. J Control Release. 2012;161(2):317–327. 22565055 10.1016/j.jconrel.2012.04.041

[4] Kraybill WG, A phase I study of fever-range whole body hyperthermia (FR-WBH) in patients with advanced solid tumours: Correlation with mouse models. Int J Hyperth. 2002;18(3):253–266. 10.1080/02656730110116704 12028640

[5] Fever Hobohm U. and cancer in perspective. Cancer Immunol Immunother. 2001;50(8):391–396. 11726133 10.1007/s002620100216 PMC11032960

[6] Hergt R, Dutz S, Müller R, Magnetic particle hyperthermia: nanoparticle magnetism and materials development for cancer therapy. J Phys Condens Matter. 2006;18:S2919–S2934.

[7] Burd R, Dziedzic TS, Xu Y, Tumor cell apoptosis, lymphocyte recruitment and tumor vascular changes are induced by low temperature, long duration (fever-like) whole body hyperthermia. J Cell Physiol. 1998;177:137–147. 9731754 10.1002/(SICI)1097-4652(199810)177:1<137::AID-JCP15>3.0.CO;2-A

[8] Mornet S, Vasseur S, Grasset F, Magnetic nanoparticle design for medical diagnosis and therapy. J Mater Chem. 2004;14(14):2161.

[9] Dewhirst MW, Viglianti BL, Lora-Michiels M, Basic principles of thermal dosimetry and thermal thresholds for tissue damage from hyperthermia. Int J Hyperthermia. 2003;19(3):267–294. 12745972 10.1080/0265673031000119006

[10] Sapareto SA, Dewey WC. Thermal dose determination in cancer therapy. Int J Radiat Oncol. Biol Phys. 1984;10(6):787–800. 6547421 10.1016/0360-3016(84)90379-1

[11] Toraya-Brown S, Sheen MR, Zhang P, Local hyperthermia treatment of tumors induces CD8(+) T cell-mediated resistance against distal and secondary tumors. Nanomedicine. 2014;10(6):1273–1285. 24566274 10.1016/j.nano.2014.01.011 PMC4119841

[12] Gandhi SJ, Minn AJ, Vonderheide RH, Awakening the immune system with radiation: optimal dose and fractionation. Cancer Lett. 2015;368(2):185–190. 25799953 10.1016/j.canlet.2015.03.024

[13] Kumari A, Simon SS, Moody TD, Immunomodulatory effects of radiation: what is next for cancer therapy? Future Oncol. 2016;12(2):239–256. 26621553 10.2217/fon.15.300 PMC5479357

[14] Dewhirst M, Viglianti BL, Lora-Michiels M, Thermal dose requirement for tissue effect: experimental and clinical findings. Proc SPIE Int Soc Opt Eng. 2003;4954:37II. 10.1117/12.476637 PMC4188373 25301982

[15] Ko EC, Forsythe K, Buckstein M, Radiobiological rationale and clinical implications of hypofractionated radiation therapy. Cancer Radiotherapie. 2011;15(3):221–229. 21514198 10.1016/j.canrad.2010.12.007

[16] Whelan TJ, Pignol J-P, Levine MN, Long-term results of hypofractionated radiation therapy for breast cancer. N Engl J Med. 2010;362(6):513–520. 20147717 10.1056/NEJMoa0906260

[17] Fogh SE, Andrews DW, Glass J, Hypofractionated stereotactic radiation therapy: An effective therapy for recurrent highgrade gliomas. J Clin Oncol. 2010;28(18):3048–3053. 20479391 10.1200/JCO.2009.25.6941 PMC2982785

[18] Formenti SC. Optimizing dose per fraction: a new chapter in the story of the abscopal effect? Int J Radiat Oncol Biol Phys. 2017;99(3):677–679. 29280462 10.1016/j.ijrobp.2017.07.028

[19] Popp I, Grosu AL, Niedermann G, Immune modulation by hypofractionated stereotactic radiation therapy: Therapeutic implications. Radiother Oncol. 2016;120(2):185–194. 27495145 10.1016/j.radonc.2016.07.013

[20] Ko EC, Benjamin KT, Formenti SC. Generating antitumor immunity by targeted radiation therapy: Role of dose and fractionation. Adv Radiat Oncol. 2018;3(4):486–493. 30370347 10.1016/j.adro.2018.08.021 PMC6200901

[21] Schaue D, Ratikan JA, Iwamoto KS, Maximizing tumor immunity with fractionated radiation. Int J Radiat Oncol Biol Phys. 2012;83(4):1306–1310. 22208977 10.1016/j.ijrobp.2011.09.049 PMC3337972

[22] Zelefsky MJ, Tumor control outcomes after hypofractionated and single-dose stereotactic image-guided intensity-modulated radiotherapy for extracranial metastases from renal cell carcinoma. Int J Radiat Oncol Biol Phys. 2012;82(5):1744–1748. 21596489 10.1016/j.ijrobp.2011.02.040 PMC4034682

[23] Johannsen M, Gneveckow U, Eckelt L, Clinical hyperthermia of prostate cancer using magnetic nanoparticles: Presentation of a new interstitial technique. Int J Hyperthermia. 2005;21(7):637–647. 16304715 10.1080/02656730500158360

[24] Maier-Hauff K, Intracranial thermotherapy using magnetic nanoparticles combined with external beam radiotherapy: results of a feasibility study on patients with glioblastoma multiforme. J Neurooncol. 2007;81(1):53–60. 16773216 10.1007/s11060-006-9195-0

[25] Johannsen M, Morbidity and quality of life during thermotherapy using magnetic nanoparticles in locally recurrent prostate cancer: results of a prospective phase I trial. Int J Hyperth. 2007;23(3):315–323. 10.1080/02656730601175479 17523023

[26] Bezman NA, Molecular definition of the identity and activation of natural killer cells. Nat Immunol. 2012;13(10):1000–1009. 22902830 10.1038/ni.2395 PMC3572860

[27] Vyas JM, Van Der Veen AG, Ploegh HL. The known unknowns of antigen processing and presentation. Nat Rev Immunol. 2008;8(8):607–618. 18641646 10.1038/nri2368 PMC2735460

[28] Dong C, Juedes AE, Temann U-A, ICOS co-stimulatory receptor is essential for T-cell activation and function. Nature. 2001;409(6816):97–101. 11343121 10.1038/35051100

[29] Elmore S Apoptosis: a review of programmed cell death. Toxicol Pathol. 2007;35(4):495–516. 17562483 10.1080/01926230701320337 PMC2117903

[30] Lanier LL. Up on the tightrope: natural killer cell activation and inhibition. Nat Immunol. 2008;9(5):495–502. 18425106 10.1038/ni1581 PMC2669298

[31] Chen L, Flies DB. Molecular mechanisms of T cell co-stimulation and co-inhibition. Nat Rev Immunol. 2013;13(7):542–542. 10.1038/nri3405 PMC3786574 23470321

[32] Yoshimura A, Naka T, Kubo M. SOCS proteins, cytokine signalling and immune regulation. Nat Rev Immunol. 2007;7(6):454–465. 17525754 10.1038/nri2093

[33] Pardo J, Bosque A, Brehm R, Apoptotic pathways are selectively activated by granzyme A and/or granzyme B in CTL-mediated target cell lysis. J Cell Biol. 2004;167(3):457–468. 15534000 10.1083/jcb.200406115 PMC2172484

[34] Akira S, Takeda K. Toll-like receptor signalling. Nat Rev Immunol. 2004;4(7):499–511. 15229469 10.1038/nri1391

[35] Trinchieri G, Sher A. Cooperation of Toll-like receptor signals in innate immune defence. Nat Rev Immunol. 2007;7(3):179–190. 17318230 10.1038/nri2038

[36] Makrilia N, Kollias A, Manolopoulos L, Cell adhesion molecules: role and clinical significance in cancer. Cancer Invest. 2009;27(10):1023–1037. 19909018 10.3109/07357900902769749

[37] Chalkley HW. Method for the quantitative morphologic analysis of tissues. J Natl Cancer Inst. 1943;4:47–53.

[38] Demidenko E Three endpoints of in vivo tumour radiobiology and their statistical estimation. Int J Radiat Biol. 2010;86(2):164–173. 20148701 10.3109/09553000903419304 PMC2900851

[39] Demidenko E Mixed models: theory and applications with R. 2nd edn. New Jersey (NJ): John Wiley & Sons, Inc.; 2013.

[40] Glasner A, Recognition and prevention of tumor metastasis by the NK receptor NKp46/NCR1. J Immunol. 2012;188(6):2509–2515. 22308311 10.4049/jimmunol.1102461

